# STATE VARIABILITY IN HEALTHCARE UTILIZATION AMONG ASSISTED LIVING RESIDENTS WITH DEMENTIA

**DOI:** 10.1093/geroni/igz038.2009

**Published:** 2019-11-08

**Authors:** Kali S Thomas, Portia Cornell, Wenhan Zhang, Momotazur Rahman, David Dosa, Brian Kaskie, Paula Carder

**Affiliations:** 1 Providence VA Medical Center, Providence, Rhode Island, United States; 2 Brown University, Providence, Rhode Island, United States; 3 Iowa State University, Aimes, Iowa, United States; 4 OHSU-PSU School of Public Health, Porland, Oregon, United States

## Abstract

The objectives of this study are to describe the state variability in the prevalence of ADRD among AL residents and in this group’s healthcare utilization. Using 2014 Medicare enrollment data, Medicare claims, and the Minimum Data Set for a sample of 493,867 Medicare fee-for-service beneficiaries in AL (178,787 with ADRD), we present their rates of hospital and emergency department (ED) use, by state, adjusting for age, sex, race, and dual-eligibility status. States varied in their unadjusted percent of residents with ADRD from 25% in MN to 48% in WV. In 2014, 40% of AL residents with an ADRD diagnosis were hospitalized during the year, ranging from 30% in UT to 47% in FL. Forty-two percent of residents with ADRD used an ED in 2014, ranging from 36% in MD to 56% in NC. The potential influence of state regulations, enforcement, and market factors will be discussed.

